# Development and Adaptation of Bilingual Health Text Message Databases for Health Interventions in Hispanic Communities: Protocol for a Scoping Review

**DOI:** 10.2196/86032

**Published:** 2026-06-24

**Authors:** Hesam A Varpaei, Susan W Buchholz, Buna Bhandari, Jennifer L Gay, Kaycee K Carmignani, Sriram Kalyanaraman, Jessica Sender, Jing Wang, Jennifer J Salinas

**Affiliations:** 1 College of Nursing Michigan State University East Lansing, MI United States; 2 Dwyer School of Health Sciences Indiana University South Bend South Bend, IN United States; 3 College of Public Health University of Georgia Athens, GA United States; 4 College of Health Sciences The University of Texas at El Paso El Paso, TX United States; 5 College of Communication Arts and Sciences Michigan State University East Lansing, MI United States; 6 MSU Libraries Michigan State University East Lansing, MI United States; 7 College of Nursing Florida State University Tallahassee, FL United States

**Keywords:** SMS, text messages, Hispanic, bilingual, health, database, review, protocol

## Abstract

**Background:**

SMS text messages have been used as part of health intervention research for over a quarter of a century. Given the ease of reaching people via SMS text message, these interventions are important for all groups, including Hispanic individuals. SMS text messaging interventions continue to show promise for improving health in this population. Several reviews have addressed aspects of designing Hispanic-focused digital health interventions. Emphasis has been placed on the linguistic and cultural relevance of SMS text messages in these studies. However, a gap exists in the literature on how SMS text messages are developed and adapted for use in health interventions with Hispanic communities across the life span.

**Objective:**

The objective of this scoping review is to answer the following question: how are bilingual SMS text message databases developed and/or adapted for use in health interventions for the Hispanic population in the United States?

**Methods:**

In this scoping review, the framework by Arksey and O’Malley and the PRISMA-ScR (Preferred Reporting Items for Systematic Reviews and Meta-Analyses extension for Scoping Reviews) guidelines will be used. The review will be conducted in five stages: (1) identifying the research question; (2) identifying relevant studies; (3) study selection; (4) charting the data; and (5) collating, summarizing, and reporting the results. For the inclusion criteria, the population (Hispanic individuals across the life span), concept (development and/or adaptation of bilingual SMS text message databases for health interventions), and context (the United States) framework will be used. There is no age or time limit on the study populations or included articles. In May 2025, we searched 4 databases: PubMed, Embase, Web of Science, and CINAHL. Covidence is being used to collect studies. There are 2 phases, each involving 2 independent reviewers: the title and abstract phase followed by the full-text review phase. One of the authors developed a data extraction tool with 4 sections. These sections include general study information, foundational SMS text message database information, how the development and adaptation of the SMS text message database were informed, and data on cultural and linguistic elements of the database. Each section will be presented in tabular format, with descriptive and numerical summaries. Inductive thematic analysis will be used for the fourth section.

**Results:**

This scoping review commenced in May 2025, and titles and abstracts are being reviewed to assess further eligibility. After the full-text review, a determination will be made on which studies to include, and data extraction and results will be documented. We anticipate that this review will be completed by December 2026.

**Conclusions:**

This scoping review of the cultural and linguistic elements of SMS text message databases may contribute to the development of more meaningful and concordant databases for use in health interventions among the Hispanic population.

**International Registered Report Identifier (IRRID):**

DERR1-10.2196/86032

## Introduction

SMS text messages have been used as part of health intervention research for over a quarter of a century [1]. SMS text messaging interventions have demonstrated effectiveness in multiple health areas, including different aspects of health promotion and chronic disease management [2]. There are multiple advantages to using SMS text messages, including their reach across populations due to widespread cellphone use. Almost all Americans (98%) have a cellphone, with 91% owning a smartphone [3].

Given the ease of reaching people via SMS text message, these interventions are important for all groups, but especially for populations at higher risk of health disparities, such as the Hispanic, Latino, or Latinx population (hereinafter referred to as “Hispanic”) [4]. Hispanic individuals are the largest racial or ethnic minority group in the United States, representing 20% of the US population [5]. In comparison to the overall US population, people who identify as Hispanic are less likely to be physically active; have higher rates of obesity, diabetes, and certain types of cancers; and are less likely to receive recommended vaccinations [6,7].

SMS text messaging interventions continue to show promise for improving health in Hispanic populations. Personalized and tailored SMS text messaging interventions have been shown to improve diabetes management by enhancing knowledge, self-efficacy, self-care, and hemoglobin A_1c_ levels among Hispanic individuals [8]. Additionally, in a study of Hispanic parent-child dyads, findings suggested that SMS text messaging is a promising tool for addressing health care disparities, reducing emergency department visits, and increasing vaccination rates [9].

Within the Hispanic population in the United States, it is important to consider how to address language for SMS text messaging interventions [10]. Among Hispanic adults in the United States, 76% report being fully proficient in English [11]. However, this remains a diverse and heterogeneous group with a broad spectrum of language preferences [12,13].

Several reviews have addressed aspects of designing health interventions, including SMS text messaging interventions, to address cultural and linguistic aspects unique to the Hispanic population. A systematic review on health literacy for US-based Spanish-speaking populations by Hernandez et al [14] recommended that SMS text messages be linguistically and culturally concordant to specific Hispanic groups. This includes addressing salient concepts and terms that are culturally relevant in regionally used Spanish. Higashi et al [15], in their scoping review of how digital health technology is used with Spanish speakers in the United States, also recommended ensuring that digital health interventions are linguistically and culturally tailored. Gonzalez et al [16] found that SMS text messaging was widely used in mobile health interventions among Hispanic communities and that there remains a need for consistent cultural tailoring. Perez Ramirez et al [17] reviewed pediatric digital health interventions in Spanish and noted that only half of the studies addressed cultural tailoring. These reviews make important contributions to the literature by demonstrating the importance of adapting language preferences in an effort to improve health interventions for the Hispanic population in the United States.

This review will contribute to the growing evidence of tailored linguistic and culturally relevant SMS text messaging interventions in current scientific literature. A gap continues to exist in the literature on how SMS text messages are developed and adapted for use in health interventions within Hispanic communities across the life span. We will conduct a scoping review to address this gap in understanding how messages are developed and adapted as core components of interventions targeted to various Hispanic populations across the United States [18,19].

## Methods

### Study Design

In this scoping review, the framework by Arksey and O’Malley [19] and the PRISMA-ScR (Preferred Reporting Items for Systematic Reviews and Meta-Analyses extension for Scoping Reviews) guidelines [20] will be used. On the basis of the framework by Arksey and O’Malley [19], the review will be conducted in the following five sequential stages: (1) identifying the research question; (2) identifying relevant studies; (3) study selection; (4) charting the data; and (5) collating, summarizing, and reporting the results. This protocol was registered in the Open Science Framework [21].

### Stage 1: Identifying the Research Question

This scoping review aims to explore the development and adaptation of SMS text message databases. While health outcomes are important in a study, assessing whether an SMS text messaging intervention is effective falls outside the parameters of this scoping review. Therefore, the review question for this scoping review is as follows: how are bilingual SMS text message databases developed and/or adapted for use in health interventions for the Hispanic population in the United States?

### Stage 2: Identifying Relevant Studies

In this scoping review, we will use the population, concept, and context framework to identify eligible studies [22].

#### Population

The population of interest is Hispanic individuals across the life span. (Study samples that include both Hispanic and non-Hispanic populations will be included if they address the concept and context of interest.)

#### Concept

The concept is the development and/or adaptation of bilingual SMS text message databases for health interventions.

#### Context

The context is the United States, where English is the official language [23]; however, Spanish is the second most spoken language in the country [24].

### Other Criteria Information

There is no age limit for the study population. There is also no time limit for studies to be included. To be included in the review, studies must meet these 3 criteria. We do not have specific exclusion criteria.

### Stage 3: Study Selection

Under the guidance of a university-based librarian, appropriate keywords were selected for the database search. These keywords were then used to search 4 online databases: PubMed, Embase, Web of Science, and CINAHL. These searches entailed 2 levels: the first being the population (eg, Hispanic, Latino, or Spanish) and the second being the type of intervention (eg, SMS text messaging and mobile health). Textbox 1 details the search that was used in this scoping review for each of the databases. This search was conducted by our university-based librarian from May 28 to 30, 2025.

We will include all study designs, including formative work and quantitative, qualitative, and mixed methods, as long as they meet the criteria of the population, concept, and context framework for our study. Eligible studies must clearly describe the process of SMS text message development and/or adaptation, including cultural and/or linguistic tailoring. Covidence (Veritas Health Innovation) [25] will be used to collect studies and determine which meet the eligibility criteria. There will be 2 screening phases: the first is the title and abstract phase followed by the full-text review phase. Initially, duplicate studies will be removed from our study pool. Next, 2 independent reviewers will screen titles and abstracts to determine whether the study meets the criteria for full-text review. In this initial screening step, studies will advance to the next screening phase if both reviewers answer “yes” to all study eligibility criteria prompts. Studies will be excluded if both reviewers answer “no” to any of the study eligibility criteria prompts. However, if there is disagreement at the initial phase, the 2 reviewers will determine whether the study should advance to the next phase. The remaining studies will then be independently reviewed by 2 researchers at the full-text phase. As with the initial screening phase, if both reviewers agree that the study continues to meet the eligibility criteria, the study will advance to the data extraction phase. If they both agree that the study does not meet the eligibility criteria upon examination of the full text, the study will not be included. A third independent reviewer will be brought in to advise on screening differences during this phase if the 2 reviewers cannot reach a consensus. Studies that continue to meet the eligibility criteria will proceed to data extraction.

To avoid bias in this scoping review, 2 authors (BB and SWB) who have published articles that could be included in this review have agreed not to review their own studies during the study selection phase. If they see at any time that one of their publications is to be reviewed, they will actively seek out 2 other reviewers to make an independent assessment of the study selection determination.

**Online databases and keywords used.**

**PubMed**

- (“Hispanic or Latino” [Mesh] OR hispanic* OR latin* OR “hispanic-american” OR “spanish-speaking” OR “spanish speaker” OR Spanish OR “latin-american” OR “latin-americans” OR “latin American” OR “latin americans”) AND (“Text Messaging” [Mesh] OR texting OR “text message” OR “text messages” OR “text messaging” OR SMS OR “short message service” OR “mobile phone message*” OR “mobile messaging”)

**Embase**

- (“hispanic”/exp OR hispanic OR latin* OR “spanish”/exp OR spanish OR “spanish-speaking” OR “spanish speaking”/exp OR “spanish speaking” OR “spanish speaker” OR “latin-american*” OR “latin american*”) AND (“texting”/exp OR texting OR “text message*” OR “text messaging”/exp OR “text messaging” OR sms OR “short message service”/exp OR “short message service”

**Web of Science**

- TS=(hispanic* OR latin* OR “hispanic american*” OR “spanish-speaking” OR “spanish speaker*” OR “latin american*” OR “latino*” OR “latina*” OR “Spanish”) AND TS=(“text message*” OR texting OR SMS OR “short message service” OR “mobile message*” OR “mobile phone message*” OR “text-based intervention*” OR “text communication” OR “mobile health” OR mHealth) AND TS=(develop* OR design* OR implement* OR deliver* OR “formative research” OR “focus group*” OR “message tailoring” OR “cultural adapt*” OR “intervention development”)

**CINAHL**

- (MH “Hispanic Americans” or Latino OR hispanic* OR latin* OR “hispanic-american” OR MH “Spanish Language” OR “spanish speaker” OR Spanish OR “latin-american” OR “latin-americans” OR “latin American” OR “latin americans”) AND (texting OR MH “Text Messaging” OR “text messages” OR “text messaging” OR SMS OR “short message service” OR “mobile phone message*” OR “mobile messaging”)

### Stage 4: Charting the Data

One reviewer will independently extract the data, and then a second reviewer will assess their completeness and accuracy.

An author-developed data extraction form will be used in this scoping review. This data extraction tool has 4 sections. The first section will extract general study information: full citation, study design, target population, sample size, setting, and health focus. The second section comprises foundational information about the SMS text message database, including origin of SMS text message database (whether the database was being developed or adapted); number of SMS text messages (in English and/or Spanish); frequency and timing of SMS text messages; database name (if available); and, if known, exemplars of how this database was used in other studies (the information supplied in this section will potentially come from other studies and will be referenced accordingly). The third section addresses elements of how the development and adaptation of the SMS text message database was informed, including use of theory, formative research, and community and stakeholder involvement. The fourth section comprises data on cultural and linguistic elements in the SMS text messages, including languages used, regional dialects, language tailoring strategy (eg, tone and selection of words to resonate with a specific group), cultural adaptation method (eg, changes made to align with the values and norms of the specific group), and personalization (eg, to a specific behavior stage).

### Stage 5: Collating, Summarizing, and Reporting the Results

Data from the included studies will be reported using the 4 data extraction sections as a guide. For the first section, descriptive and numerical summaries will be used to present the general characteristics of the included studies, such as publication year, country, study design, target population, sample size, setting, and health focus. These data will be presented in tabular format, providing a high-level overview of the scope and distribution of the evidence. For the second section, descriptive and numerical summaries will be provided, with data documented in a tabular format to show the origin of the SMS text message database, any adaptations to the database, the number of SMS text messages, the frequency and timing with which those SMS text messages are intended to be used or have been used, and the name of the database. If known, exemplars of how the database has been used will be provided. For the third section, descriptive and numerical summaries will be used to present data in tabular form on how theory, formative research, and/or community and stakeholder involvement occurred during the development and adaptation of the SMS text message database. For the fourth section, the data will initially be presented in a tabular format. However, with these data, which document the cultural and linguistic elements of the SMS text messages, an inductive thematic analysis will be undertaken to assess how themes emerge directly from the data without a preexisting theoretical constraint [26].

It is acknowledged that not all fields may contain complete or directly comparable information. In such cases, necessary modifications will be made to accommodate the available data, and some fields may remain unpopulated across the documentation of multiple studies.

## Results

This scoping review commenced in May 2025. After duplicates were removed, 2531 titles and abstracts were reviewed to assess whether they were eligible for full-text review. After this initial review, 255 full-text articles are now being reviewed to determine which studies meet the eligibility criteria. Once this is completed, data extraction will be completed. Results will then be synthesized. We anticipate that this review will be completed by December 2026. Conception of the scoping review was developed by a team of researchers working on the 50K4Life study (NCT06411769; J Salinas: principal investigator).

## Discussion

### Expected Findings

The strengths of this scoping review are the addition to the literature of a comprehensive review of the development and adaptation of bilingual SMS text message databases for the Hispanic population. It is anticipated that data on the linguistic and cultural adaptation of SMS text message databases will be available based on prior literature reviews that emphasize the importance of ensuring that health interventions are appropriately tailored to specific Hispanic population groups [14,16]. Researchers can then determine whether linguistic and cultural adaptation is sufficient or whether further work is needed before including a specific SMS text message database in a bilingual intervention. In addition, researchers will be able to compare databases by reason for development (potentially, if available as a bilingual database) for work on health promotion (eg, increasing physical activity and healthy eating), prevention (eg, vaccination and smoking cessation), population health (eg, infant health, breastfeeding, and caregivers), disease (eg, diabetes, asthma, and mental health), health management (eg, medication adherence and health care use), and other possible SMS text message databases [1,2,27].

The limitations of this review are that there may be no published work on the development and adaptation of bilingual databases, and therefore, we will not be able to include it in this review or the possibility that those bilingual databases simply do not exist. Contributing to this is the limitation that the search strategy does not include gray literature and, therefore, may not locate posters, presentations, or unpublished studies. Additionally, while the review may inform the development of future interventions, this study will not assess outcomes and cannot comment on whether the methods identified are successful.

This review will provide a foundational starting point for SMS text message development for other researchers (or a way for them to continue their work). This will allow research teams to determine whether they want to incorporate any of those findings and, if not, why, along with consideration of bringing in other development aspects that may not have been widely used. Researchers will have an opportunity to review how existing bilingual databases have been developed and potentially adapted over time. This scoping review of the cultural and linguistic elements of those databases will potentially contribute to more meaningful, concordant, and effective SMS text message databases for use in health interventions.

### Conclusions

This scoping review will examine how bilingual SMS text message databases have been developed and potentially adapted for use in health interventions among the Hispanic population. Findings from this review may inform the development of future interventions and guide researchers and practitioners in designing culturally responsive health communication strategies.

## Abbreviations

PRISMA-ScR: Preferred Reporting Items for Systematic Reviews and Meta-Analyses extension for Scoping Reviews

## Data Availability

Data sharing is not applicable to this paper as no datasets were generated or analyzed during this study.
